# Comparison of various flow maldistribution quantification methods in mini heat exchangers

**DOI:** 10.1038/s41598-023-38784-5

**Published:** 2023-07-17

**Authors:** Paweł Dąbrowski

**Affiliations:** Faculty of Mechanical Engineering and Ship Technology, Institute of Energy, Gdańsk University of Technology, Narutowicza 11/12, 80-233 Gdańsk, Poland

**Keywords:** Mechanical engineering, Energy science and technology

## Abstract

The aim of study is to compare various flow maldistribution quantification methods, using velocity, mass flow rate, pressure, and temperature. An experimentally validated numerical study has been prepared and a heat exchanger with 34 semi-circular channels with a diameter of 3.1 mm has been tested. The minichannels were heated from the bottom with a heat flux of 50, 60, 70, and 80 kW/m^2^. The cases for various inlet velocities of 0.1, 0.2, 0.3, and 0.4 m/s have been tested. It results in a total of 16 cases with various heat flux and various inlet velocities of the water. Then, for every 16 cases, the flow maldistribution coefficients, widely used in the literature, have been calculated based on the velocity, pressure, and temperature profiles. The study shows that every method gives other results of the same parameter that should define the flow distribution in the heat exchanger in the same way. Hence, the ambiguities of fluid distribution conclusions in heat exchangers that can be found in the literature may be caused by a different interpretation of the flow maldistribution coefficient. A normalized flow maldistribution coefficient that gives the same results for all thermohydraulic parameters used has been proposed.

## Introduction

Tuckerman and Pease^1^ proved that reducing the hydraulic diameter of the channel results in a more intense heat transfer. Since then, many researchers deal with mini or microchannels^2,3^. However, as is well known the smaller the hydraulic diameter of the channel the higher the velocity (for constant mass flow rate) hence the higher the pressure drop. To maintain the pressure drops of the flow in the heat exchanger at a reasonable level, one can divide the total mass flow rate into many branches. It results in lowering the velocity in every path (while maintaining the total mass flow rate constant) and hence reduce the pressure drop. However, many minichannels connected with common inlet and outlet manifolds provide another hydraulic issue, namely the uneven distribution^4^. This phenomenon is called most often flow maldistribution and creates problems not only in heat exchangers^5^ but also in other technology areas e.g. gas desulfurization towers^6^ or fuel cells^7^.

However, uneven flow may be desirable and there are applications (e.g. in chemical engineering when designing a reaction in a reactor^8^ or in cooling electronics where heterogeneous heat flux on the surface can be encountered^9^) where non-uniform distribution is used intentionally to improve the overall performance of the device. Li et al.^10^ showed that the flow in parallel minichannel heat sinks can be tailored (different mass flow rates in every channel) to eliminate the temperature hotspots when non-uniform multiple-peak heat flux occurs. Hence, the flow maldistribution (“bad/incorrect/wrongful” distribution) does not necessarily mean non-uniform distribution and at the same time, the non-uniformly distributed flow is not always a maldistribution. Nevertheless, in this paper, the assumption is that favorable fluid distribution is equivalent to a uniform velocity and temperature distribution, so the terms "flow maldistribution" and "non-uniform flow" are used interchangeably here.

In the last years, a few reviews concerning the flow maldistribution in mini, micro, compact and macrochannels heat exchangers have been published^11–14^. Dario et al.^11^ focused on the two-phase flow distribution in parallel channels where factors influencing the two-phase flow maldistribution and some header designs which improve the flow uniformity have been discussed. Siddiqui and Zubair^12^ discussed the flow maldistribution from the heat exchanger’s geometry (mainly manifolds) point of view. The authors showed some analytical models that tried to mathematically describe the flow maldistribution. Ghani et al.^13^ focused mostly on the manifold design in the scope of the flow maldistribution. The authors discussed deeply what manifolds can be distinguished and how particular manifolds influence fluid distribution. Singh et al.^14^ described the flow maldistribution in several heat exchangers (plate, plate-fin, and tube-fin) and show the effect of phase change and properties variations on the flow distribution. The authors place their work in the context of solar collectors and more sustainable energy utilization.

The novelty of the study is the comprehensive summary of previous works on the flow maldistribution and the comparison between results that seem often to be ambiguous is a significant contribution. The comparison of results from various works in terms of recalculating it using different methods of flow maldistribution quantification has not been found in the literature before. The summary shows that ambiguities that can be found in the literature may be caused by a different interpretation of the flow maldistribution coefficient. The prepared numerical analysis validated with the experiment shows that the best and universal flow maldistribution coefficient can be found thanks to the statistical data analysis.

## Flow maldistribution quantification methods

In the literature, several methods to quantify the flow maldistribution can be found. For this purpose, some characteristic parameter (mass flow rate, velocity, pressure drop, temperature) that can be distinguished in a given channel of the heat exchanger is determined and it refers to the appropriate value that should be in each channel if the flow is completely uniform.

### Velocity measurements

One of the methods of quantitative analysis of the flow non-uniformity is to determine the velocity field on the heat exchanger surface. If the minichannel heat exchanger is taken into account, then the determination of the velocity in each of the parallel channels will allow determining the mass flow rate in each of them, which allows describing the phenomenon of flow maldistribution. Calculating the mass flow rate on the base of the velocity in the channels is possible only when the cross-sectional areas of each of them are known. When quantifying flow maldistribution by velocity, it is often assumed that each channel has the same cross-section. This is true in most cases, but this assumption should be always in mind. If the channels have different cross-sectional areas, the non-uniform velocity field on the heat exchanger surface does not necessarily mean that there is a non-uniform mass flow rate. Kumar et al.^15,16^ carried out numerical studies in which they showed that by changing the width or height (cross-sectional area) of the individual channels it is possible to eliminate the flow maldistribution.

Kim et al.^17^ conducted an experiment in which the velocity in parallel channels was measured to determine the flow maldistribution in a minichannel heat exchanger. Channels with a semicircular cross-section (radius 1.55 mm) made of aluminum were used. To be able to visualize the flow and measure the velocity in the channels, the measuring section was covered with acrylic glass. Red ink soluble in water was introduced periodically into the water flowing in the section. Thanks to this procedure, it was possible to observe the boundary of water without dye and red-dyed water. This border was followed by a high-speed camera that filmed the flow at 200 frames per second. After dividing the distance covered by the border of colored and dyed water visible in the photo by the time needed to cover it, the authors obtained the velocity in each minichannel. The tests were carried out in the range of the volumetric flow rate from 3.33 to 6.67 cm^3^/s with the I’-type flow configuration. The expression in Eq. (1) was used to determine the flow maldistribution coefficient in every channel.

1 $$\Phi_{i} = \frac{{U_{\max } - U_{i} }}{{U_{avg} }}$$

In this case, the average velocity U_avg_ is the velocity that would occur in all the channels if the flow were completely uniform. The authors found that the highest velocity occurs in the central channels and the lowest in the side channels, which is consistent with the observations of other authors^15,16^. Moreover, greater flow maldistribution occurs with higher flow rates and when the ratio of the channels’ width to their length increases. The distance between the heat exchanger inlet and the channels also influences the distribution of the working medium. The higher it is, the more uniform the flow becomes, which was also observed by other researchers^18^. Furthermore, authors^17^ proposed an analytically derived equation for the maldistribution coefficient which involves the geometrical parameters of a heat exchanger (width, channel length, channel spacing, channel’s hydraulic diameter, and channels distance from the inlet). It corresponds quite well with the authors’ results. However, the authors stressed that this theory is limited in the assumption of uniform spreading.

A slightly different approach to the flow maldistribution coefficient calculation was presented by Minqiang et al.^18^ or Dąbrowski et al.^19^. To be able to present it quantitatively, they used Eq. (2).

2 $$\Phi = \sqrt {\frac{1}{N}\sum\limits_{i = 1}^{N} {\left( {\frac{{U_{i} - U_{avg} }}{{U_{avg} }}} \right)}^{2} } \times 100\%$$

The above equation allows the calculation of the aforementioned coefficient for the entire heat exchanger, and not only for individual channels, as in Eq. (1). This is a useful approach as it enables different heat exchanger designs to be compared with each other, e.g., with a different number of channels. The authors numerically verified the dependence of the length, height, and width of the channels, the location of the inlet of the working medium, as well as the distance between individual channels on the flow maldistribution phenomenon.

### Flow rate measurements

Another method of quantitative analysis of flow maldistribution is the measurement of the mass flow rate. Such an approach to the issue of fluid distribution makes it possible to become independent of the influence of unequal cross-sectional areas of parallel channels on the correct interpretation of the phenomenon. It is the mass flow rate at a given point in the heat exchanger that directly affects its operation, including the distribution of the temperature field, pressure drop, and the efficiency of thermal energy transport.

Individual measurement of the mass flow in each of the 25 parallel channels was carried out by Kumaraguruparan et al.^20^. The authors conducted an experiment and a numerical simulation for water. To estimate the quantitative distribution of the flow maldistribution in the minichannel heat exchanger, the water passing through the set of channels was collected individually for each minichannel without the use of an outlet manifold. The flow maldistribution coefficient for the entire heat exchanger was calculated using Eq. (3).

3 $$\Phi = \frac{{\dot{m}_{\max } - \dot{m}_{\min } }}{{\dot{m}_{avg} }} \times 100\%$$

The minimum, maximum, and average values refer to the mass flow in individual channels. Based on the conducted research^20^, the authors concluded that two types of pressure drops can be distinguished: related to inertia forces (velocity decrease, and thus pressure increase) and related to friction forces. To reduce the flow maldistribution, it is necessary to reduce the influence of inertia and increase the influence of friction. Higher flow rates deteriorate fluid distribution. Numerical studies have shown the presence of flow separation, backflows, and swirls at the inlet to the channels, and they are the ones that cause uneven distribution. The authors say that these effects can be counteracted by increasing the viscosity of the fluid. In addition, a greater pressure drop should be in the channels themselves.

The flow maldistribution coefficient can also be presented in two stages. Two expressions are used for this^21,22^. The first one, presented in Eq. (4), is used to determine the coefficient in each minichannel.

4 $$\Phi_{i} = \sqrt {\left( {\frac{{\dot{m}_{i} - \dot{m}_{avg} }}{{\dot{m}_{avg} }}} \right)^{2} }$$

To determine it, the mass flow rate at the point and the average value of the mass flow rate that would be in each channel if the flow were uniform are needed. Most often it is the total mass flow flowing through the heat exchanger divided by the number of channels. The flow maldistribution coefficient equal to zero indicates an ideal flow, and the higher it is, the more the mass flow in the channel (point) under consideration differs from the average. After determining the flow maldistribution coefficient for all channels, the standard deviation is determined to calculate the total coefficient for the entire heat exchanger (Eq. 5).

5 $$\Phi = \frac{{\sum\limits_{i = 1}^{N} {\Phi_{i} } }}{\sqrt N } \times 100\%$$

Thanks to it, different heat exchangers can be compared with each other, differing in design, number of channels, or the shape of the manifold, without the need to analyze changes in the flow maldistribution coefficient in individual channels. Instead, one characteristic value can be used.

### Pressure drop measurements

In addition to measuring the flow rate or velocity in individual channels, another method for quantifying the flow maldistribution is to measure pressure drops in the channels. Pressure is one of the hydrodynamic parameters of the flowing fluid and is directly related to the flow velocity, e.g., the Bernoulli equation or Navier–Stokes equations^23^. For this reason, the difference in pressure drop in individual channels can be treated as an indicator of the quality of fluid distribution. This approach has been used by several researchers.

Siva et al.^24^ presented in their work experimental and numerical studies describing flow maldistribution in minichannels. The experimental tests were carried out for the flow of water to which heat was supplied. The maximum heat flux was 50 kW/m^2^. Variable parameters that were analyzed in terms of their impact on the flow maldistribution were:

- the hydraulic diameter of the channels (88, 176, or 352 µm)
- number of channels (5, 10, or 20)
- Reynolds number (from 10 to 200)
- the ratio of the cross-sectional area of the channels and manifolds (0.5 or 2)
- the flow configuration (U-type, Z-type, or I-type)

To quantify the flow maldistribution, the authors^24^ measured the inlet and outlet pressures of each channel. Thanks to these measurements, it was possible to calculate the pressure drops in individual channels. The flow maldistribution coefficient was defined as the ratio of the maximum and minimum pressure drop difference to the maximum pressure drop, according to Eq. (6).

6 $$\Phi = \frac{{\Delta p_{\max } - \Delta p_{\min } }}{{\Delta p_{\max } }} \times 100\%$$

Research has shown that the greater the number of channels, the worse the fluid distribution. Moreover, the flow configuration has a great influence on the maldistribution coefficient. The worst distribution was visible for the U-type and the best for the I-type. When the ratio of the manifold’s cross-sectional area to the channels’ cross-sectional area decreases, a greater flow maldistribution can be observed. Thanks to numerical studies, the authors concluded that the non-uniformity of the flow and the non-uniform temperature field on the heat exchanger surface is closely related. In addition, special attention should be paid to the verification of numerical calculations. Often the results of CFD calculations are of poor quality and therefore may not match the experiment. This may be because, in the case of minichannels, viscous forces dominate over inertia forces, which is often not taken into account in numerical calculations.

Another work, using Eq. (6) to calculate the flow maldistribution coefficient was presented by Maganti et al.^25^. In this work, numerical calculations were taken into account for the liquid flow with nanoparticles in 7 parallel minichannels (hydraulic diameter of 100 µm). The flow was realized in the U-type configuration, and the ratio of the channels' cross-sectional area and the manifold was 0.2. The authors described the distribution of particles as well as the fluid and their effect on thermal parameters. It was observed that the flow distribution is more uneven with large heat flux and low Reynolds number. As the heat flux increases, the temperature increases, and the viscosity of the fluid decreases, and thus the influence of viscous forces decreases. At the same time, for smaller Reynolds numbers, the flow velocity decreases with a constant hydraulic diameter. In the previously described studies^17,20^, the decrease in the flow velocity increased the flow maldistribution coefficient. This is an inaccuracy that recurs in the literature and is one of the reasons why the described phenomenon should still be investigated. This discrepancy may result from the range of tested parameters, the method of calculating the flow maldistribution coefficient, or the type of flow (single-phase, two-phase).

In later studies, Maganti et al.^26^ also used pressure measurement to determine the flow distribution in 7, 10, or 12 parallel minichannels with a hydraulic diameter of 100 or 200 µm. The Reynolds number range over which the experiment was carried out was from 10 to 150, and the input heat flux was 2 or 5 kW/m^2^. Three types of flow configuration were tested: U-type, I-type, and Z-type. The ratio of the channels' cross-sectional area and the manifold was equal to 0.2 as before. The authors claimed that the worst fluid distribution is noticeable for the U-type flow, which is consistent with the studies of other authors^24^. On the other hand, the best uniformity according to Maganti et al.^26^ is guaranteed by Z-type flow configuration, which contradicts the reports from Siva et al.^24^. However, the differences in the flow maldistribution coefficient between Z-type and I-type are insignificant, so they can be caused by measurement inaccuracies. Additionally, the authors^26^ found that although the flow maldistribution is due to hydrodynamics, the study of this phenomenon only under adiabatic conditions is of very little importance. It is the temperature distribution under heat transfer conditions that plays a key role in the design of cooling systems. The viscosity of the fluid is a function of temperature, and the non-uniformity of the flow depends on the viscous forces, therefore examining the temperature field provides a better insight into the actual design and operation of the heat exchangers.

### Temperature profile measurements

Another method of testing the flow maldistribution is to determine the temperature distribution on the heat exchanger surface. The simplest solution is to use a thermal imaging camera to determine the temperature field and analyze it for the presence of points of increased temperature and the shape of isotherms. For a completely uniform distribution, assuming a constant heat flux over the entire surface of the heat exchanger, the isotherms should be straight lines, perpendicular to the direction of the fluid flow. The analysis of the temperature field allows for the qualitative determination of the non-uniformity. When applying heat to a fluid, its higher temperature means that the mass flow is lower there, and the lower the temperature, the opposite. However, it does not allow quantifying the mass flow rate or the velocity of the fluid. To do this, it is necessary to include in the considerations the aspect of heat transfer and the temperature difference between the fluid and wall that forces this exchange.

Li and Hrnjak^27^ developed a method to quantify the mass flow distribution of the liquid refrigerant in a minichannel heat exchanger from images recorded with an infrared camera. This method is based on assuming a relationship between the mass flow rate of the fluid in each minichannel and the thermal efficiency on the side of the second heat transfer fluid, calculated based on the wall temperature measurement. The authors verified their method based on numerical calculations and experimental research. The tests were carried out on the evaporator of the cooling system. The evaporator's cooling capacity is mainly due to the latent heat of the liquid refrigerant, so the liquid distribution is more important than the vapor distribution.

The quantitative analysis of the flow maldistribution based on the temperature field was carried out by Paz et al.^28^. They recorded images taken with a thermal imaging camera on an evaporator operating in the ORC (Organic Rankine Cycle) installation^29,30^. Ethanol was the fluid flowing through the system. The research has shown that it is possible to compare the temperature fields and on their basis determine whether the working medium distributes uniformly or not.

Numerical studies of ten different configurations of a minichannel heat exchanger to find a configuration resulting in maximum thermal efficiency and uniform temperature distribution were carried out by Vasilev et al.^31^. To compare quantitatively the uniformity of the temperature field on the heat exchanger surface, the authors introduced the coefficient presented in Eq. (7).

7 $$\Phi_{q} = \frac{{T_{\max } - T_{\min } }}{q}$$

It was found that at low velocities of the medium flowing in the channels, and thus also at low Reynolds numbers, a more significant dependence of the temperature distribution uniformity on the number of direction changes during the flow in the channel can be observed. In addition, it was noted that the more uniform temperature distribution contributed to an additional increase in the mean Nusselt number for the same pumping power.

### Comparison

As can be seen in the above considerations, the flow maldistribution can be quantified in many different ways, using various types of parameters, such as velocity, mass flow rate, volume flow rate, pressure drop, or temperature. The authors researched flow maldistribution phenomena using different experimental techniques as well as numerical simulations. Moreover, the geometries tested in terms of flow distribution differ in many aspects, e.g. number of channels, flow configuration, or mass flow rate. Hence, it is difficult to compare the results. Besides, some authors^24,26^ noticed that the worst fluid distribution is for the U-type flow while the others^32^ that the V-type results in the highest flow maldistribution. Even severer incoherence can be found when considering fluid flow rate as a maldistribution parameter. There are authors^25^ that claimed less uniform distribution is for low flow rates number while some authors^17,20^ claimed the opposite.

To make the comparison easier and to explain mentioned ambiguities the results from different studies have been recalculated using various flow maldistribution quantifying methods. The flow maldistribution coefficient was calculated taking into account velocity, mass flow rate, pressure drop, and temperature distribution using Eqs. (2), (3), (6), and (7) respectively. The studies were selected in a way that has enough data to calculate at least 3 of 4 maldistribution coefficients. The results were shown in Table 1. The data was sorted from the lowest to the highest channels’ hydraulic diameter tested. All the data were taken for the basic, conventional cases even if a particular paper presented the geometry that would lower the flow maldistribution coefficient.

**Table 1:** Comparison of flow maldistribution coefficient calculated in various ways.

Authors	N	D_h_ (µm)	Re_ch_ (–)	$\dot{m}$ (kg/s)	$q$ (kW/m^2^)	Φ – velocity (%)	Φ – pressure drop (%)	Φ – mass flow rate (%)	Φ_q_ – temperature [(K m^2^)/MW]
Xia et al.^33^	30	150	309–615	1.67e−3–3.33e−3	2000	99.4	–	213.3	6.0 (high flow rate)
						–	–	–	8.6 (low flow rate)
Kumar et al.^15^	25	315	134–269	1.00e−3–2.00e−3	1000	38.8 (low flow rate)	–	85.0 (low flow rate)	40.5 (high flow rate)
						54.0 (high flow rate)	–	111.3 (high flow rate)	53.0 (low flow rate)
Kumar and Singh^22^	28	1500	165	8.33e−3	150	11.8	–	40.3	140
Kumar and Singh^9^	28	1500	165	8.33e−3	200	26.6 (I)	–	68.9 (I)	267.5 (I)
						27.1 (U)	–	80.7 (U)	273.5 (N)
						30.7 (N)	–	114.3 (Z)	292.5 (U)
						34.5 (Z)	–	117.6 (N)	299.7 (Z)
Song et al.^34^	20	3000	947	6.83e−2	150	27.3 (trapezoidal I)	63.9 (rectangular)	82.0 (trapezoidal I)	143.3 (rectangular)
						30.9 (rectangular)	66.7(trapezoidal I)	85.0 (rectangular)	156.7 (trapezoidal I)
			474–1412	3.41e−2–1.02e−1	70–150	21.2 (low flow rate)	–	–	116.7 (high flow rate)
						36.0 (high flow rate)	–	–	192.9 (low flow rate)

Firstly, it can be concluded that every flow maldistribution coefficient differs depending on the equation chosen even if the data used describe the same results. Hence, the flow maldistribution coefficient cannot be easily compared without making sure that they were calculated using the same equation. Moreover, in every case, the flow maldistribution coefficient calculated using Eq. (3) is several times higher than the flow maldistribution coefficient calculated using Eq. (2) for the same data. Furthermore, it is worth noticing that the flow maldistribution coefficient calculated by Eq. (7) is not a percentage value. It has its dimension and varies significantly depending on heat flux, so it cannot be compared easily.

Secondly, the flow maldistribution coefficient is higher for high flow rates when considering velocity (Eq. 2) and mass flow rate (Eq. 3). Nonetheless, the coefficient for the same cases but calculated using temperature profile (Eq. 7) is higher for low flow rates. That is a possible reason for results incoherencies in a manner of flow maldistribution and flow rates. Studies that would allow making a comparison of the flow maldistribution coefficient calculated using Eq. (6) with other coefficients for high and low flow rates have not been found.

It can be also seen that the worst flow configuration is also ambiguous. The results from Kumar and Singh^9^ show that the best flow uniformity is obtained when the I-type flow configuration is implemented. However, the rest of the tested flow configurations' order is not the same for every flow maldistribution coefficient. Lastly, the differences between the flow maldistribution coefficients for various manifold shapes are not significant.

## CFD model details

In the numerical simulation, the minichannel heat exchanger based on the experiment from Kim et al.^17^ has been taken into account. The three-dimensional model was created with the ANSYS SpaceClaim software and the fluid domain was prepared. The working medium was assumed as water. The fluid in channels flows in the X-axis direction. The water was heated up from the bottom of the section with a constant heat flux (in the Z-axis direction). The material of the heat exchanger was assumed as aluminum. The inlet and outlet manifolds were not heated up. The whole heat was transported to the minichannels.

**Figure 1:**
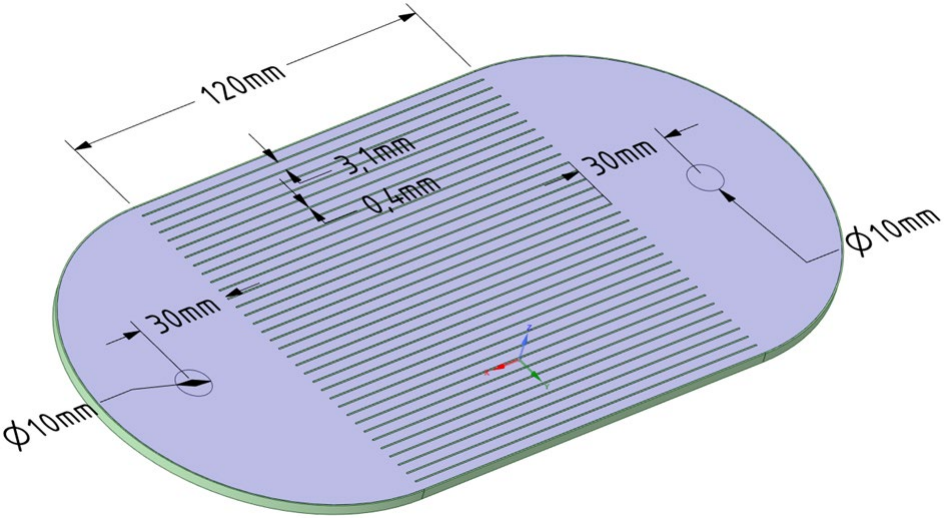
Physical model of minichannel heat exchanger used in the numerical simulation.

The physical model of a minichannel heat exchanger with 34 semi-circular channels with a diameter of 3.1 mm and a length of 120 mm has been shown in Fig. 1. To investigate the behavior of various maldistribution coefficients in some range of flow rates, the cases for various inlet velocities of 0.1, 0.2, 0.3, and 0.4 m/s have been tested. It results in a total of 16 cases with various heat fluxes and various inlet velocities of the water. Then, for every 16 cases, the flow maldistribution coefficients, widely used in the literature, have been calculated based on the velocity, pressure, and temperature profiles in the minichannel heat exchanger.

### Governing equations and boundary conditions

During the numerical simulations, the following assumptions were taken:

- Properties of fluid were independent of pressure and dependent on temperature.
- Fluid flow was a single-phase, steady-state, incompressible, and three-dimensional.

The continuity, momentum, and energy equations (Eqs. 8, 9, and 10) were taken into account as governing equations and used in calculations together with the above-mentioned assumptions.

8 $$\nabla \cdot \overrightarrow {U} = 0$$

9 $$\rho (\overrightarrow {U} \cdot \nabla \overrightarrow {U} ) = - \nabla p + \mu \nabla^{2} U$$

10 $$\rho C_{p} (\overrightarrow {U} \cdot \nabla T) = k\nabla^{2} T$$

The gravity acceleration was neglected. The specific heat C_p_, thermal conductivity k, density ρ and dynamic viscosity µ of water were dependent on temperature according to polynomials with coefficients shown in Table 2. The polynomials were created in a temperature range of 274.15 K–372.15 K based on NIST Standard Reference Database 23^35^.

**Table 2:** The coefficients a, b, c, d, e, and f for polynomials describing temperature dependency of specific heat C_p_, thermal conductivity k, density ρ, and dynamic viscosity µ.

	aT^5^	bT^4^	cT^3^	dT^2^	eT^1^	fT^0^
C_p_	−4.594153e−8	7.580797e−5	−5.00605e−2	1.654468e1	−2.737428e3	1.856145e5
k	−6.329234e−12	1.083902e−8	−7.388976e−6	2.496865e−3	−4.157021e−1	2.768943e1
ρ	1.490802e−9	−2.498934e−6	1.685605e−3	−5.747475e−1	9.896856e1	−5.859364e3
µ	−5.775095e−13	9.474075e−10	−6.229481e−7	2.053244e−4	−3.394845e−2	2.255142

In ANSYS FLUENT 2021 R1 the conservation equations of mass, momentum, and energy are solved using the finite volume method (FVM). Momentum and energy equations are discretized by the second-order upwind scheme. In this case, model SST k-omega has been chosen as a turbulence model. Before final calculations, 3 main and most common models have been tested: laminar, SST k-omega, and k-epsilon. The best results in terms of convergence and consistency with the experimental/correlation results have been obtained for the SST k-omega model. As known, this model gives good results near a wall, which is desirable in small channels, and good results in bigger volume, which is desirable in bigger manifolds^36–39^. A segregated implicit solver with SIMPLE pressure correction algorithm has been chosen to compute the velocity field in the whole heat exchanger (inlet/outlet manifolds and minichannel section).

Water was chosen as a working fluid for all the considered cases. The inlet temperature for water is T = 300 K and the parameters for aluminum are ρ = 2719 kg/m^3^, C_p_ = 871 J/(kg K), k = 202.4 W/(m K). The heat was applied at the bottom wall of a section with a constant value of heat flux q of 50, 60, 70, and 80 kW/m^2^. The constant velocity at the inlet to the heat exchanger was equal to 0.1, 0.2, 0.3, and 0.4 m/s which result in Reynolds number at the inlet of 995, 1990, 2986, and 3981 respectively. The average velocity U_avg_ in a single minichannel was 0.061, 0.122, 0.184, and 0.245 m/s, and the mean Reynolds number in a single channel was 115, 230, 345, and 460 respectively. The pressure-outlet boundary condition was assumed at the outlet of the heat exchanger. When the residual values become less than 10^−3^ for the continuity, x-velocity, y-velocity, and z-velocity and 10^−6^ for the energy the solutions are considered to be converged.

### Model validation

**Figure 2:**
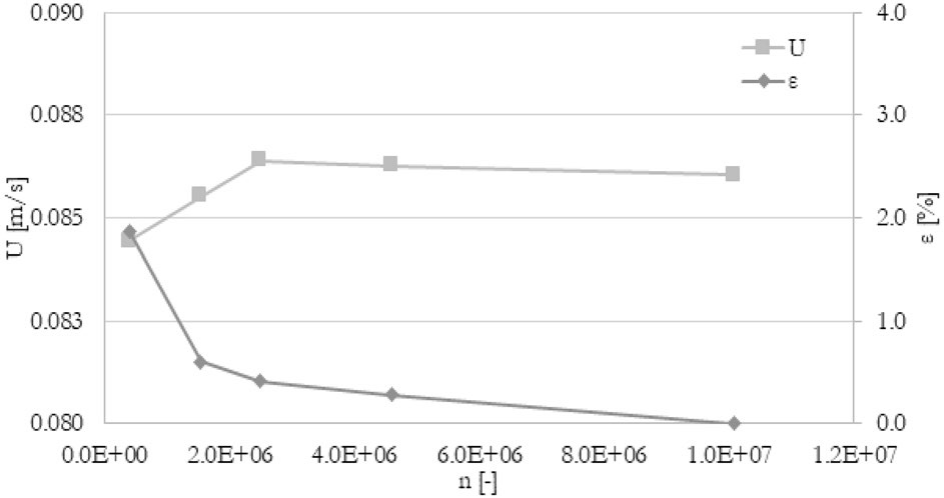
The results of the grid independence study. U—the average velocity at the outlet, ε—percentage difference of velocity.

**Figure 3:**
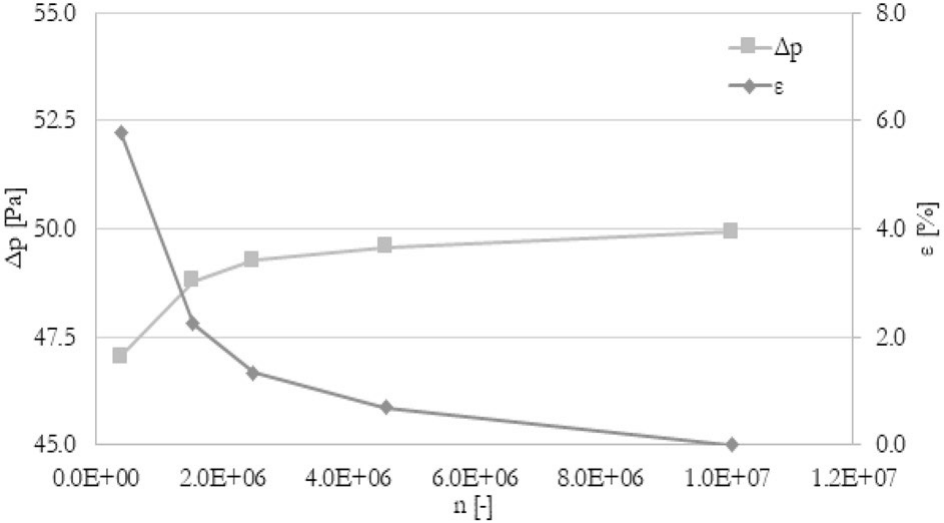
The results of the grid independence study. Δp—the pressure drop at the entire heat exchanger, ε—percentage difference of pressure drop.

**Figure 4:**
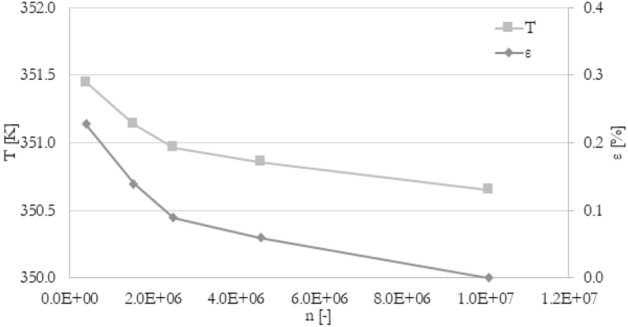
The results of the grid independence study. T—the average temperature in the minichannels, ε—percentage difference of temperature.

The mesh independence study was carried out to ensure the accuracy of numerical results. The procedure was similar to^40^. There were five different meshes tested with a various number of elements: from 4.0e5 elements to 1.0e7 elements. To compare various meshes, the percentage deviation ε of tested parameter F (velocity, pressure drop, temperature) between j-th mesh and the finest mesh has been introduced (Eq. 11). The mesh for which the absolute percentage deviation is less than 1% for outlet velocity, pressure drop, and the average temperature in channels has been chosen for all simulations. Mesh independence study, as well as experiment validation, has been prepared for the following boundary conditions: 120 mm long minichannels, an inlet flow rate of 3.33e−6 m^3^/s (200 ml/min), and heat flux of 50 kW/m^2^ at the bottom wall. The results of velocity, pressure drop, and temperature were shown in Figs. 2, 3, and 4, respectively. The mesh with about 4.6e6 elements has been chosen for further calculations.

11 $$\varepsilon = \left| {\frac{{F_{j} - F_{fine} }}{{F_{fine} }}} \right| \times 100\%$$

**Figure 5:**
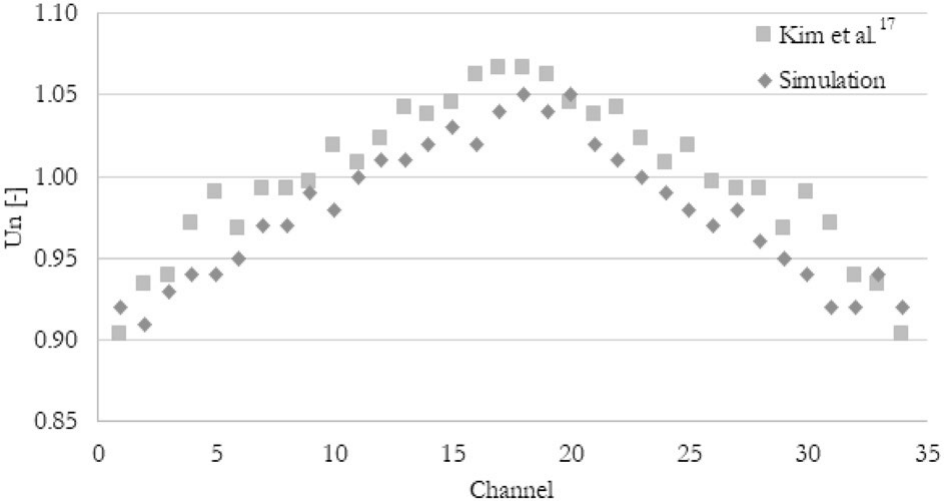
The comparison of normalized velocity in the minichannel section for current simulation and experiment from Kim et al.^17^.

Thanks to the use of the exact geometry as in experimental work from Kim et al.^17^, the current simulation could be validated with the experimental data. To do so, the normalized velocity U_n_ was introduced and its mathematical expression has been shown in Eq. (12). The normalized velocity from the experiment and the simulation has been shown in Fig. 5. The results show good agreement, especially taking into account that there is an uncertainty in measurements. However, there is no uncertainty analysis in the work from Kim et al.^17^ so the uncertainties could not be marked.

12 $$U_{n,i} = \frac{{U_{i} }}{{U_{avg} }}$$

**Figure 6:**
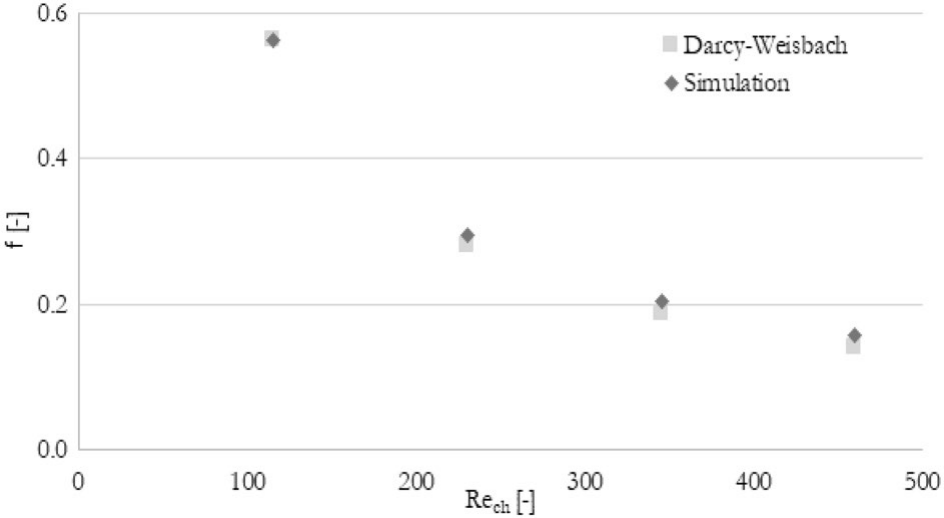
The comparison of average friction factor in minichannels for current simulation and Darcy–Weisbach correlation.

Moreover, the current numerical model has been verified by comparing values of the simulated friction factor (Eq. 13) in the minichannel section with the correlation of Darcy–Weisbach (Eq. 14). Results of the comparative analysis of friction factor number are shown in Fig. 6. A good agreement between the current model and theoretical correlation ensures the accuracy and reliability of the current numerical analysis.

13 $$f = \frac{{2D_{h} \Delta p}}{{L\rho U^{2} }}$$

14 $$f_{D - W} = \frac{64}{{\text{Re}}}$$

## Results and discussion

From the above literature review^18,20–22,24^, it can be concluded that to quantify the flow maldistribution, average velocity, mass flow rate, pressure drop, or temperature drop (rise) in channels should be taken into account. Worth to notice, that in this particular study, velocity distribution matches the mass flow rate distribution due to constant channels’ cross-sections Moreover, four types of equations that describe the flow maldistribution in the entire heat exchanger can be distinguished. However, not every method has been seen in the literature for every thermohydraulic parameter. To present all dependencies more consistently, a parameter F, which corresponds to average velocity, pressure drop, or temperature drop in channels has been introduced. Equation (15) takes into account a difference between a maximum value of parameter F in channels and the value of parameter F in the i-th channel referred to the average value (if the flow would be completely even) of parameter F. Equation (16) takes into account a difference between the average value of parameter F and value of parameter F in i-th channel referred to the average value of parameter F. Equation (17) takes into account a difference between the maximum value of parameter F in channels and minimum value of parameter F in channels referred to the average value of parameter F. Equation (18) takes into account a difference between the maximum value of parameter F in channels and minimum value of parameter F in channels referred to a maximum value of parameter F in channels. In the following considerations, the flow maldistribution coefficient with subscripts U, p, and T will refer to one based on the velocity, pressure drop, and temperature drop as a parameter F respectively. Moreover, subscripts 1, 2, 3, and 4 will refer to the flow maldistribution coefficient calculated from Eqs. (15), (16), (17), and (18) respectively.

15 $$\Phi_{1} = \sqrt {\frac{{\sum\limits_{i = 1}^{N} {\left( {\frac{{F_{\max } - F_{i} }}{{F_{avg,ch} }}} \right)}^{2} }}{N}} \times 100\%$$

16 $$\Phi_{2} = \sqrt {\frac{{\sum\limits_{i = 1}^{N} {\left( {\frac{{F_{avg,ch} - F_{i} }}{{F_{avg,ch} }}} \right)}^{2} }}{N}} \times 100\%$$

17 $$\Phi_{3} = \frac{{F_{\max } - F_{\min } }}{{F_{avg,ch} }} \times 100\%$$

18 $$\Phi_{4} = \frac{{F_{\max } - F_{\min } }}{{F_{\max } }} \times 100\%$$

The velocity, pressure, and temperature fields are used interchangeably to quantify the fluid distribution in heat exchangers not without a reason. Those thermohydraulic parameters are connected. To show this connection, not only velocity but also pressure drop in a single channel and temperature drop (rise) in a single channel have been normalized in a similar way as in Eq. (12). The pressure drop in a single channel, so the pressure difference between the inlet and outlet of the particular channel has been divided by the average pressure drop in all channels. The same has been done with the temperature drop, so the temperature difference between the inlet and outlet of the particular channel.

**Figure 7:**
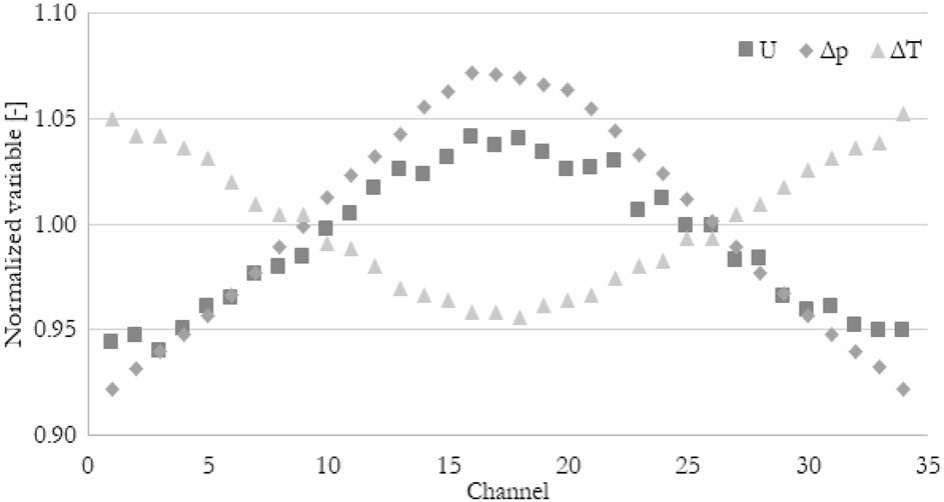
Exemplary normalized velocity, normalized pressure drop, and normalized temperature drop distribution in minichannels for Re_ch_ of 115 and heat flux of 80 kW/m^2^.

**Figure 8:**
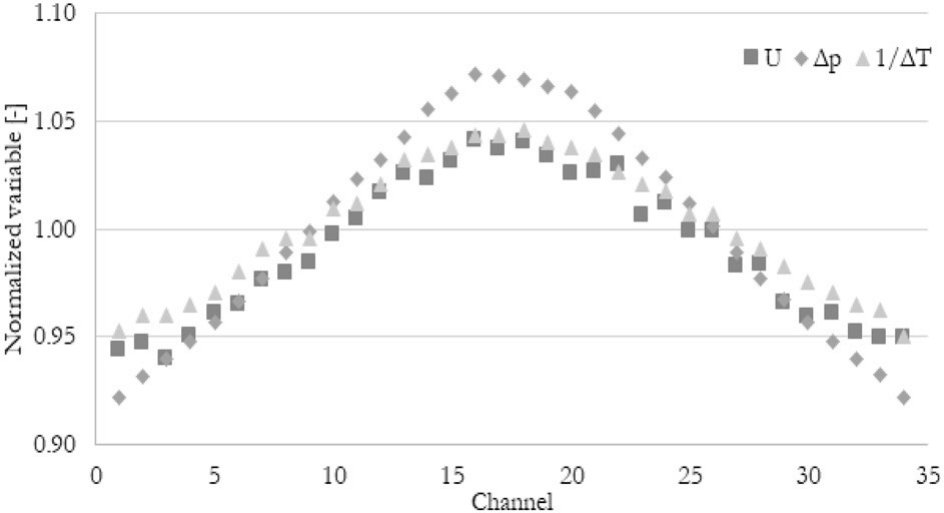
Exemplary normalized velocity, normalized pressure drop, and inverse normalized temperature drop distribution in minichannels for Re_ch_ of 115 and heat flux of 80 kW/m^2^.

The normalized thermohydraulic parameters distribution for case Re_ch_ of 115 and heat flux of 80 kW/m^2^ has been shown in Fig. 7. For the rest of the cases, the distribution is qualitatively the same. As can be seen, velocity and pressure drop distributions are almost the same. The temperature drop distribution in minichannels is the opposite of the others because the higher the velocity (higher flow rate) in a given channel, the smaller the temperature increase due to the constant heat flux at the wall. To visualize that all the 3 parameters behave in the same way, the inversed normalized temperature drop has been introduced, which is shown in Fig. 8. Now it can be seen that mentioned thermohydraulic parameters distributions should give the same (or very similar) results in terms of flow maldistribution coefficient, no matter which parameter (velocity, pressure, temperature) has been taken into account.

### Various parameters

**Figure 9:**
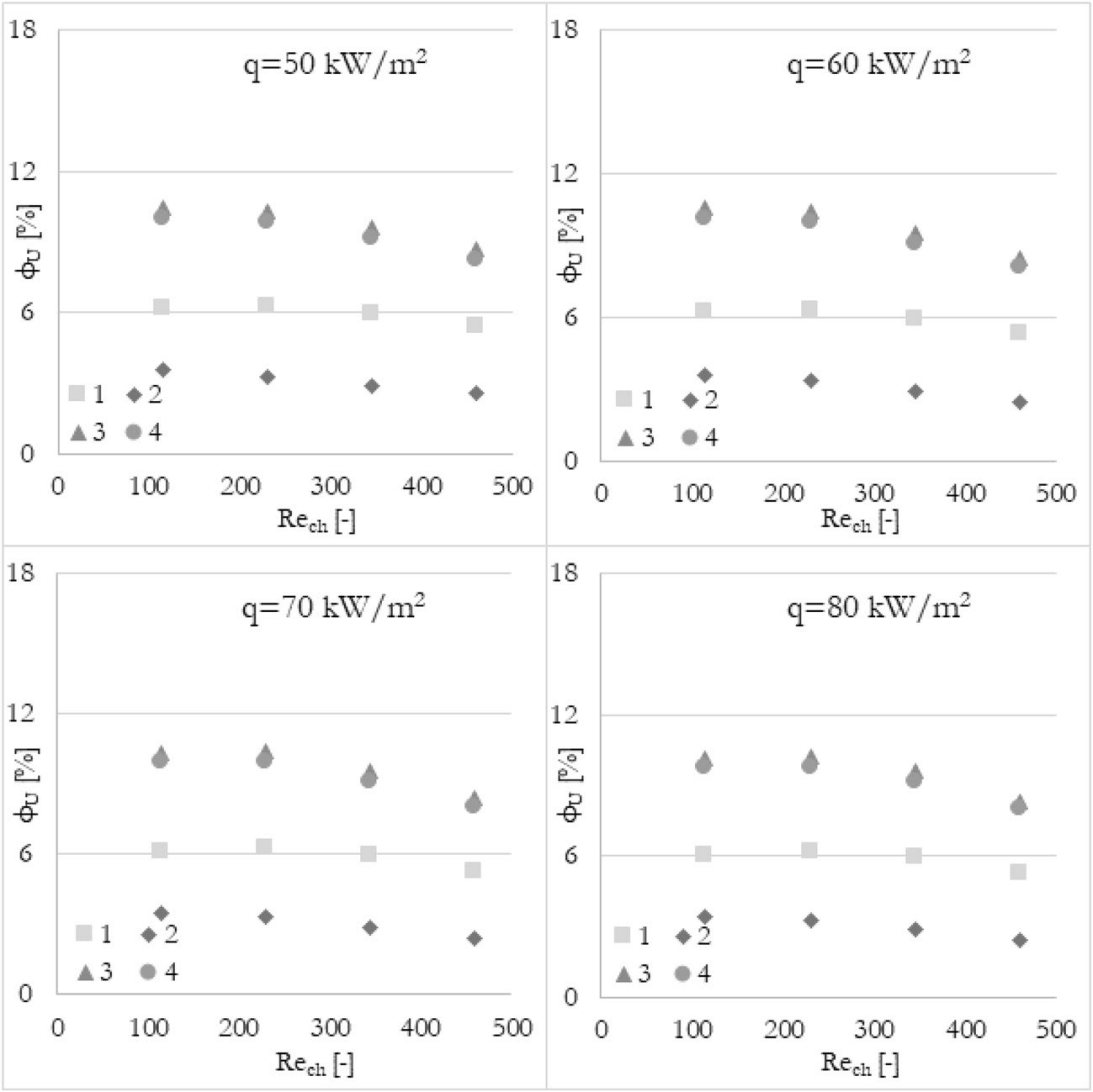
Flow maldistribution coefficient calculated using velocity and various methods in function of average Reynolds number in channels Re_ch_ for various heat fluxes q.

**Figure 10:**
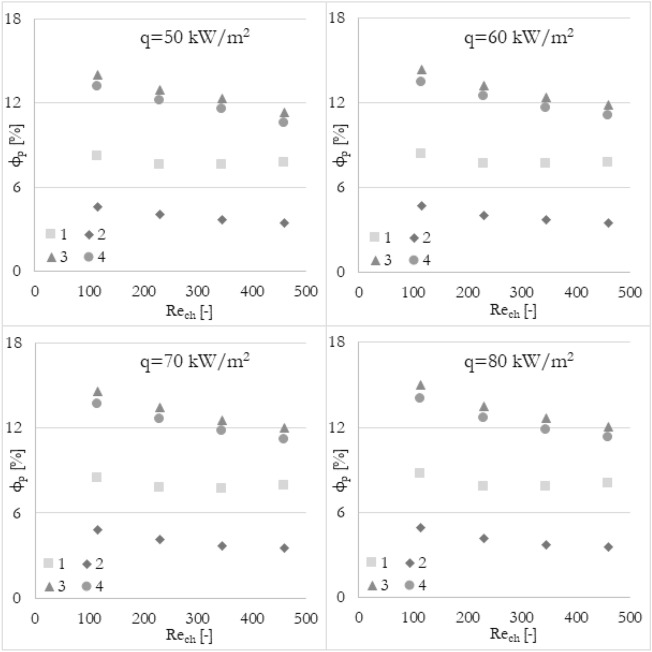
Flow maldistribution coefficient calculated using pressure and various methods in function of average Reynolds number in channels Re_ch_ for various heat fluxes q.

**Figure 11:**
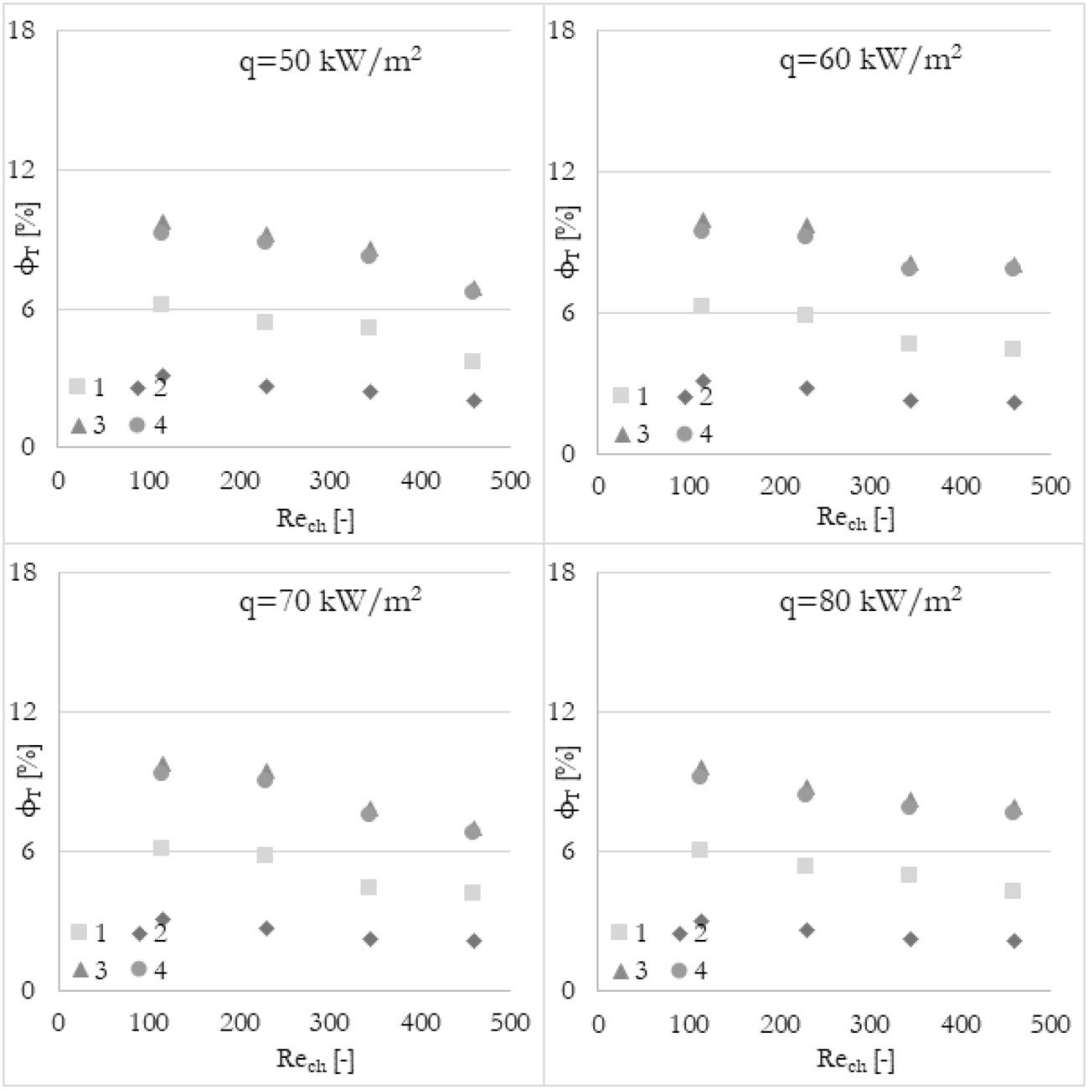
Flow maldistribution coefficient calculated using temperature and various methods in function of average Reynolds number in channels Re_ch_ for various heat fluxes q.

Firstly, it has been analyzed how the flow maldistribution coefficient (calculated in various ways) is dependent on the Reynolds number (the inlet velocity in this particular case) and heat flux applied to the wall surface. To show the dependence, data has been grouped into 3 sets of graphs (Figs. 9, 10, and 11) where every set corresponds to the flow maldistribution coefficient calculated using a particular thermohydraulic parameter (velocity, pressure, or temperature). In every set, there are 4 graphs and each of them shows data for a particular heat flux. Moreover, every graph contains 4 series of data that correspond to various methods used to calculate a flow maldistribution coefficient (Eqs. 15–18).

In Fig. 9 the flow maldistribution coefficient utilizing velocity profile for calculation has been shown. It can be seen that irrespectively of the chosen calculation method, the flow distribution is better for higher Reynolds numbers. It can be seen that the highest dependence can be noticed for methods 3 and 4 while similar, small dependence of Reynolds number is for methods 1 and 2. Moreover, there is no significant difference between flow maldistribution coefficients for different heat fluxes.

In Fig. 10 the flow maldistribution coefficient utilizing pressure profile for calculation has been shown. It can be seen that for most calculation methods, the flow distribution is better for higher Reynolds numbers. However, for method 1 the flow maldistribution coefficient is almost constant and seems to be not correlated linearly (correlation coefficient of 0.448). The rest of the methods used show a high linear correlation coefficient with Reynolds number, namely 0.912, 0.951, and 0.961 for methods 2, 3, and 4 respectively. The highest linear dependence can be noticed for methods 3 and 4 with tanα of about −0.008, while for method 2 is about −0.004. Moreover, there is no significant difference between flow maldistribution coefficients for different heat fluxes.

In Fig. 11 flow maldistribution coefficient utilizing the temperature profile for calculation has been shown. Again, it can be seen that for every calculation method, the flow distribution is better for higher Reynolds numbers. For all methods, the linear correlation coefficient is about 0.960 or more. Still, the highest linear dependence of flow distribution with Reynolds number can be noticed for methods 1, 3, and 4 with tanα of about −0.005, while for method 2 tanα is about −0.003.

The common conclusion for all data is that the highest values of the flow maldistribution coefficient are for methods 3 and 4 and the lowest for method 2. Moreover, methods 3 and 4 give always very similar results and are the most influenced by the Reynolds number.

### Various methods

**Figure 12:**
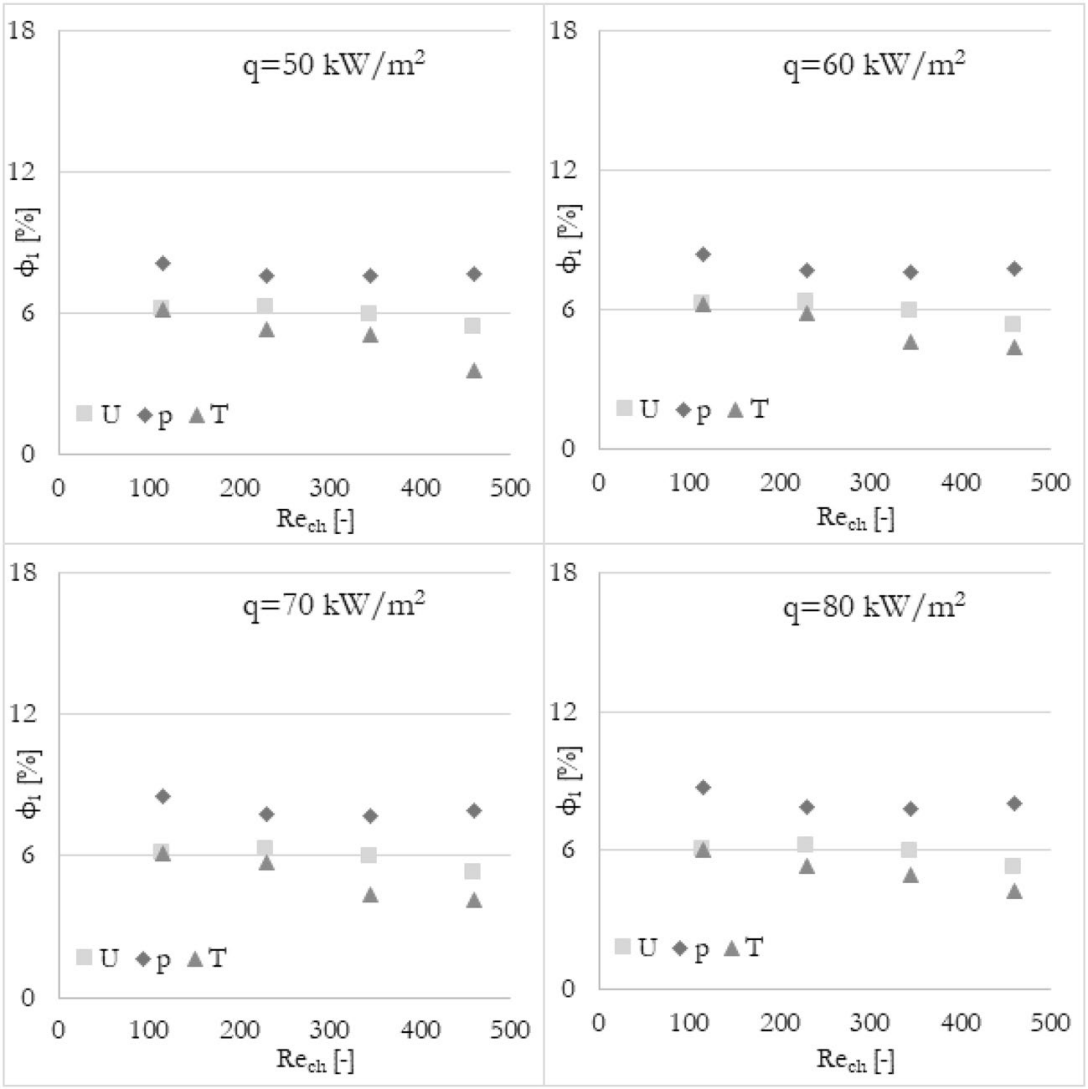
Flow maldistribution coefficient calculated using Eq. (15) and various parameters in function of average Reynolds number in channels Re_ch_ for various heat fluxes q.

**Figure 13:**
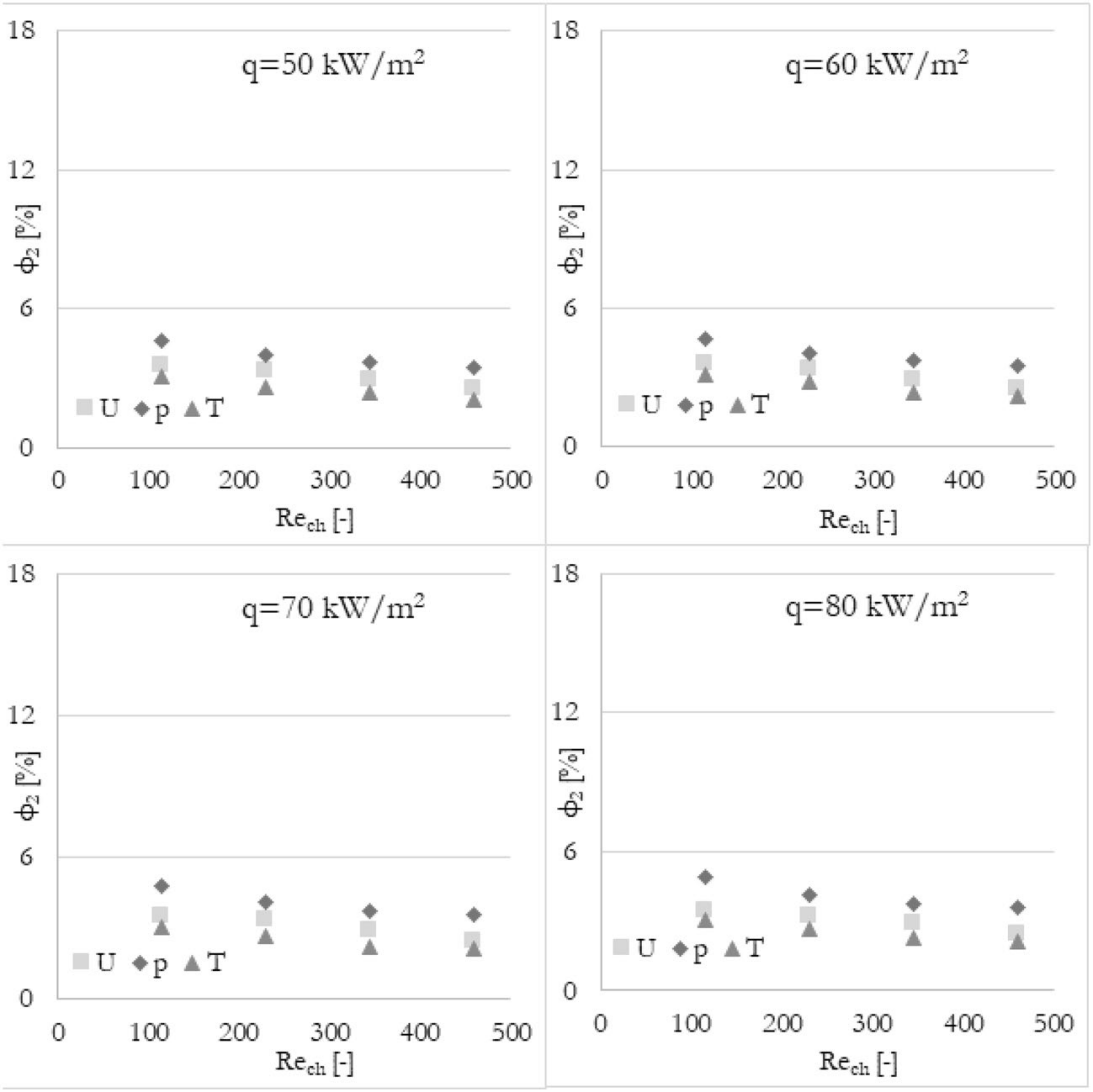
Flow maldistribution coefficient calculated using Eq. (16) and various parameters in function of average Reynolds number in channels Re_ch_ for various heat fluxes q.

**Figure 14:**
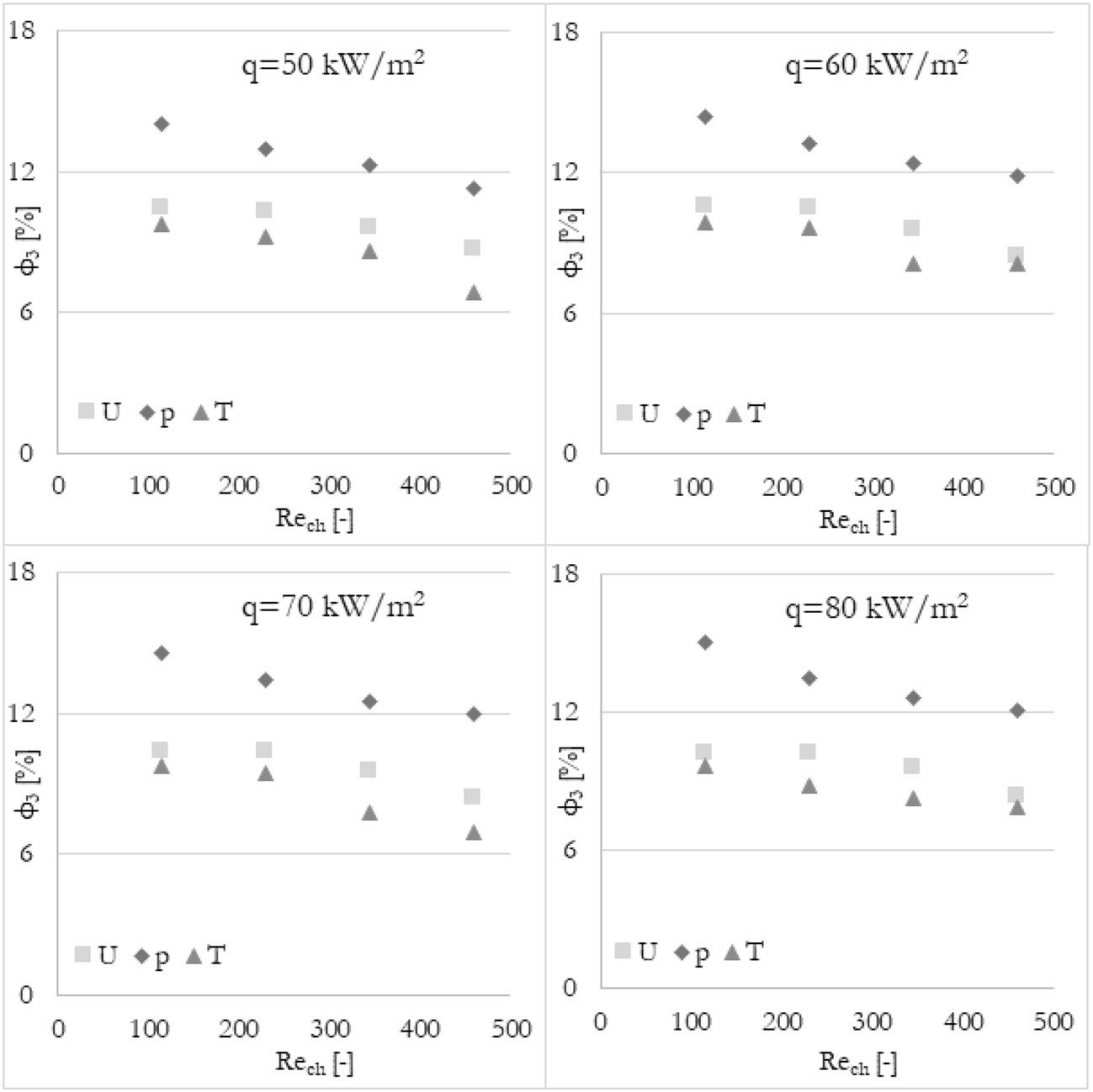
Flow maldistribution coefficient calculated using Eq. (17) and various parameters in function of average Reynolds number in channels Re_ch_ for various heat fluxes q.

**Figure 15:**
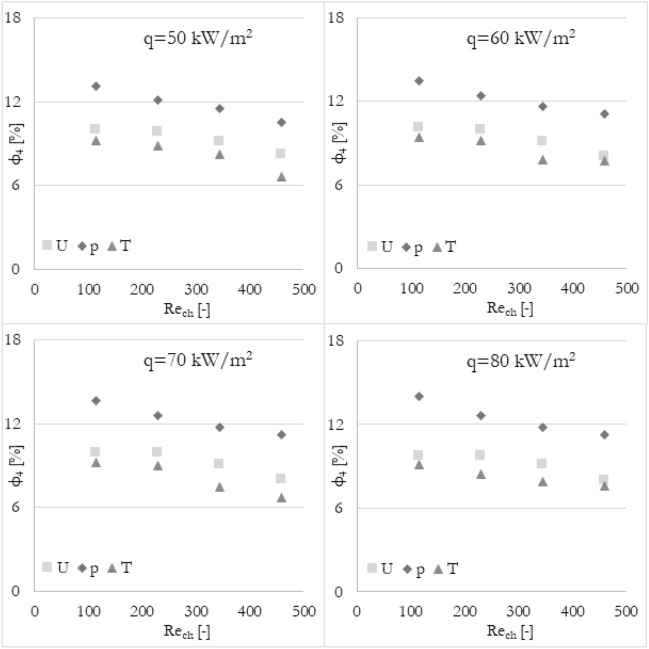
Flow maldistribution coefficient calculated using Eq. (18) and various parameters in function of average Reynolds number in channels Re_ch_ for various heat fluxes q.

Furthermore, it has been analyzed how the various calculation methods of flow maldistribution coefficient work with different thermohydraulic parameters that were taken into account for distribution analysis. Looking at Fig. 8, it can be deduced that no matter what thermohydraulic parameter is taken into account, the same (or very similar) results, in terms of flow maldistribution, should be obtained. Once again, the flow maldistribution coefficient (calculated in various ways) has been shown in the function of the Reynolds number for various heat fluxes applied to the wall surface. To look at the same data from a different perspective, the results have been grouped into 4 sets of graphs (Figs. 12, 13, 14, and 15) where every set corresponds to the flow maldistribution coefficient calculated using a particular method (Eqs. 15–18). In every set, there are 4 graphs and each of them shows data for a particular heat flux. Moreover, every graph contains 3 series of data that correspond to the various thermohydraulic parameter (velocity, pressure, and temperature) used to calculate a flow maldistribution coefficient.

In Fig. 12 the flow maldistribution coefficient utilizing method 1 (Eq. 15) for calculation has been shown. It can be seen that the values of the flow maldistribution coefficient for various thermohydraulic parameters are close to each other but the differences between them are rising with a higher Reynolds number. The average values of tanα for velocity, pressure, and temperature functions are −0.0023, −0.0019, and −0.0049 respectively, so the dependence of flow distribution and Reynolds number is different when various thermohydraulic parameter is taken into account.

In Fig. 13 the flow maldistribution coefficient utilizing method 2 (Eq. 16) for calculation has been shown. It can be seen that the values of the flow maldistribution coefficient for various thermohydraulic parameters are close to each other for a whole range of Reynolds numbers. Moreover, this method seems to be constant for various heat fluxes even when using temperature as a calculating parameter. The average values of tanα for velocity, pressure, and temperature functions are −0.0029, −0.0039, and −0.0026 respectively, so the dependence of flow distribution and Reynolds number is very similar for various thermohydraulic parameters.

In Fig. 14 the flow maldistribution coefficient utilizing method 3 (Eq. 17) for calculation has been shown. It can be seen that the differences between particular flow maldistribution coefficient values are significant. It can also be observed that the flow maldistribution coefficient utilizing temperature as a thermohydraulic parameter is changing not linearly with the Reynolds number, which is inconsistent with most cases. Moreover, the average values of tanα for velocity, pressure, and temperature functions are −0.0060, −0.0078, and −0.0072 respectively.

In Fig. 15 the flow maldistribution coefficient utilizing method 4 (Eq. 18) for calculation has been shown. It can be seen that the differences between particular flow maldistribution coefficient values and main trends and conclusions are similar to method 3. Method 4 utilizing temperature as a calculating parameter is dependent on the heat flux significantly. The average values of tanα for velocity, pressure, and temperature functions are very similar to that in method 3, namely −0.0056, −0.0073, and −0.0062 respectively.

### Quantitative comparison

To compare various methods of calculating the flow maldistribution coefficient using various parameters quantitatively, the standard deviation for every method (k = 1, 2, 3, or 4) has been introduced according to Eqs. (19) and (20). It shows if a particular method gives a similar numerical value describing a flow distribution for every parameter (velocity, pressure, or temperature) used. According to the previous analysis, a good indicator should show the same (or very similar) value no matter what parameter has been used for calculation. Low standard deviation means that the particular flow maldistribution coefficient does not show many differences between several thermohydraulic parameters.

19 $$\sigma_{k} = \frac{{(\Phi_{U,k} - \Phi_{avg,k} )^{2} + (\Phi_{p,k} - \Phi_{avg,k} )^{2} + (\Phi_{T,k} - \Phi_{avg,k} )^{2} }}{3}$$

20 $$\Phi_{avg,k} = \frac{{\Phi_{U,k} + \Phi_{p,k} + \Phi_{T,k} }}{3}$$

Since the flow maldistribution coefficient is already a percentage value, a unit of standard deviation should be interpreted as a percentage point (pp).

**Figure 16:**
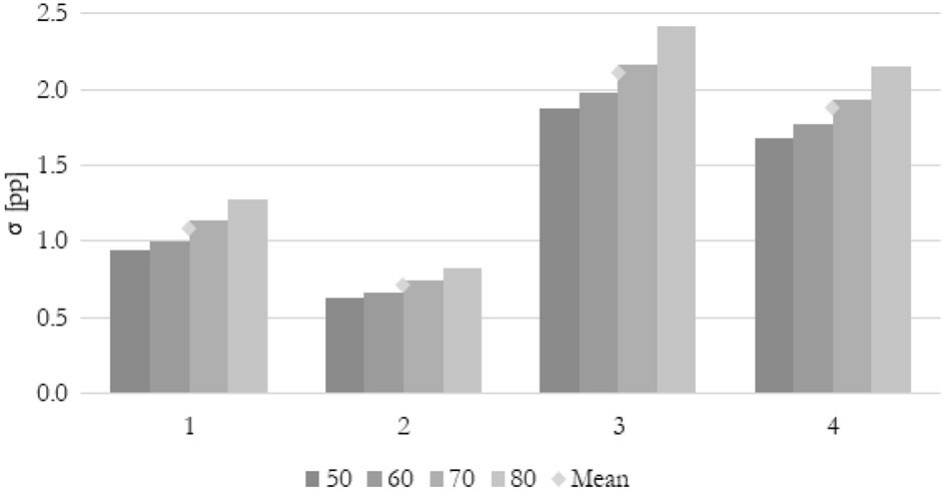
The standard deviation of maldistribution coefficient between various thermohydraulic parameters for various calculation methods, average Reynolds number in channels Re_ch_ of 115, and heat flux q of 50, 60, 70, and 80 kW/m^2^.

In Fig. 16 the standard deviation of the maldistribution coefficient between velocity, pressure, and temperature chosen as a calculating thermohydraulic parameter for Re_ch_ of 115 and changing heat flux is shown. In this graphical representation of data, all the above considerations can be concluded. The lowest mean standard deviation of 0.58 pp is for method 2. Other methods show significantly higher standard deviation. Moreover, method 2 is the only one that’s standard deviation is almost equal throughout the entire range of the heat flux.

**Figure 17:**
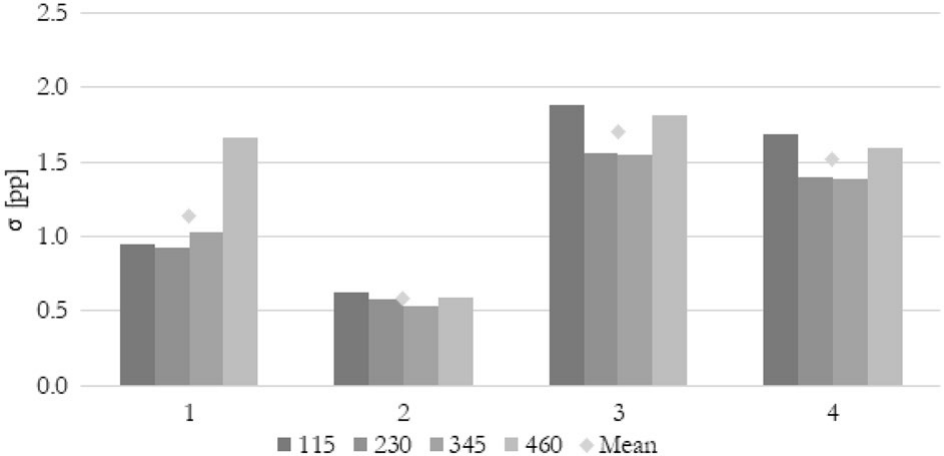
The standard deviation of maldistribution coefficient between various thermohydraulic parameters for various calculation methods, heat flux q of 50 kW/m^2^, and average Reynolds number in channels Re_ch_ of 115, 230, 345, and 460.

In Fig. 17 the standard deviation of the maldistribution coefficient between velocity, pressure, and temperature chosen as a calculating thermohydraulic parameter for q of 50 kW/m^2^ and changing Reynolds number is shown. It can be seen that the standard deviation for methods 1, 3, and 4 is very sensitive to the Reynolds number. The good flow distribution indicator should give the same results for all thermohydraulic parameters no matter of inlet velocity of the working fluid. The only method that seems to be independent of these changes is method 2. Moreover, it gives the lowest mean standard deviation of 0.58 pp.

## Conclusions

In summary, the phenomenon of flow maldistribution is the subject of research by scientists from around the world. The main attention is focused on the distribution of the fluid in sets of parallel minichannels, connected by common inlet and outlet manifolds. As described before, there are many methods to quantify the flow maldistribution. Nevertheless, various methods use various thermohydraulic parameters and compare minimum, maximum, or average values to each other. Moreover, some methods are used only for pressure distribution while others use temperature or velocity. The variety of methods is big and creates inconsistencies in the conclusions. Also, the variety of the flow maldistribution coefficients results in difficulties in comparing the values from different studies. The flow maldistribution coefficient differs depending on the equation chosen even if the data used describe the same results.

In current studies, the most common flow distribution quantification methods have been compared using all thermohydraulic parameters for every method described according to Eqs. (15)–(18). This approach allowed us to see how the particular method works with the particular thermohydraulic parameter. During the analysis, it has been concluded that the best flow maldistribution coefficient should generate the same result for every thermohydraulic parameter. Moreover, it should not be influenced by heat flux because the temperature profile is an effect of flow distribution and not a cause.

The best quantitative indicator of flow distribution in terms of stable results for all thermohydraulic parameters is method 2 presented in Eq. (16). It can be simplified to the form presented in Eq. (21). The normalized flow maldistribution coefficient can take into account any normalized thermohydraulic parameter F_n_ (normalized velocity in channels, normalized pressure drop in channels, normalized temperature drop (rise) in channels). The normalized thermohydraulic parameter in an i-th channel can be defined with Eq. (22).

21 $$\Phi_{n} = \sqrt {\frac{{\sum\limits_{i = 1}^{N} {\left( {1 - F_{n,i} } \right)}^{2} }}{N}} \times 100\%$$

22 $$F_{n,i} = \frac{{F_{i} }}{{F_{avg} }}$$

## Data Availability

All data included in this study are available upon request by contact with the corresponding author.

## List of symbols

C_p_: Specific heat, J/(kg K)
D_h_: Hydraulic diameter, m
f: Friction factor (–)
F: Tested parameter (velocity, pressure drop, temperature)
k: Thermal conductivity, W/(m K)
L: Channel length, m
ṁ: Mass flow rate, kg/s
n: Number of mesh elements (–)
N: Number channels (–)
pp: Percentage point (–)
q: Heat flux, W/m^2^
Re: Reynolds number (–)
T: Temperature, K
U: Velocity, m/s

## Greek symbols

α: Inclination angle to the abscissa axis, rad
Δp: Pressure drop, Pa
ΔT: Temperature drop, K
ε: Percentage deviation, %
µ: Dynamic viscosity, Pa s
ρ: Density, kg/m^3^
σ: Standard deviation, percentage point
Φ: Flow maldistribution coefficient, %
Φ_q_: Thermal flow maldistribution coefficient, (m^2^ K)/W

## Subscripts

avg: Average
ch: In channel
D-W: According to the Darcy–Weisbach correlation
fine: The finest mesh
i: In i-th channel
j: For j-th mesh
k: For k-th method (equation)
max: Maximum
min: Minimum
n: Normalized
p: Calculated using pressure
T: Calculated using temperature
U: Calculated using velocity

## Abbreviation

ORC: Organic Rankine Cycle
